# Prospective study on health-related quality of life in patients before and after cochlear implantation

**DOI:** 10.1007/s00405-021-06631-w

**Published:** 2021-02-09

**Authors:** Michaela Plath, Theresa Marienfeld, Matthias Sand, Philipp S. van de Weyer, Mark Praetorius, Peter K. Plinkert, Ingo Baumann, Karim Zaoui

**Affiliations:** 1 Department of Otorhinolaryngology, Head and Neck Surgery, University Hospital Heidelberg, Ruprecht-Karls-University, Im Neuenheimer Feld 400, 69120 Heidelberg, Germany; 2 GESIS-Leibniz-Institute for the Social Sciences, Mannheim, Germany; 3 Department of Otorhinolaryngology, Head and Neck Surgery, University Hospital Hamburg, Hamburg, Germany

**Keywords:** HRQoL, Cochlear implant, NCIQ, APHAB, HPS, Prospective data

## Abstract

**Purpose:**

Assessing cochlear implant (CI)-associated patient outcomes is a focus of implant research. Most studies have analyzed outcomes retrospectively with low patient numbers and few measurement time points. In addition, standardized CI-specific health-related quality of life (HRQoL) instruments have not been used. To address this, we prospectively assessed HRQoL in patients before and after implantation.

**Methods:**

We assessed HRQoL using the Nijmegen Cochlear Implant Questionnaire (NCIQ), Abbreviated Profile of Hearing Aid Benefit (APHAB), Hearing Participation Scale (HPS), and the Visual Analogue Scale (VAS) in 100 deaf or severely hearing-impaired patients (57 unilaterally deaf and 43 bilaterally deaf) before and 3, 6, and 12 months after cochlear implantation. We compared the results of unilaterally and bilaterally hearing-impaired patients and patients with or without a hearing aid. Principal component (PCA) and exploratory factor analyses (EFA) were also conducted.

**Results:**

The NCIQ measured improvements in all 6 domains after CI and correlated well with other QoL instruments. The PCA revealed that the NCIQ can be better explained by physical, physical advanced, and socio-psychological components. The APHAB score ameliorated over time, except for the background noise domain. The overall HPS score improved over time, but the hearing handicap subscore significantly decreased. Sociodemographic influences on the questionnaire scores were relatively weak.

**Conclusion:**

Assessing HRQoL is essential for quantifying the patient outcome after CI. NCIQ scores in our patient cohort showed improved HRQoL in all domains and we recommend that the NCIQ be used as a first-line questionnaire for assessing QoL in hearing-impaired patients after CI.

## Introduction

Hearing loss is the third most common chronic condition in adults and has serious health-related implications [1–3]. Fewer relationships, decreased social activity, and increased depression are the psycho-social consequences of untreated hearing loss [4, 5]. Cochlear implants (CI) can restore hearing in people with severe or total hearing loss when hearing aids have little or no effect [6]. They may also improve speech recognition and quality of life (QoL) [1, 7–13]. However, the quality of studies on this topic is low, and the type and frequency of complications and side effects have not been investigated [6]. In recent years, research focus has shifted to the health-related quality of life (HRQoL) after cochlear implantation. Some studies have retrospectively analyzed improvements in hearing ability, speech perception, and speech production [14–17], but cochlear implantation also affects the social life and self-esteem of patients [18]. So far, there is no standardized questionnaire for assessing HRQoL after cochlear implantation [19, 20]. Authors have used either the Nijmegen Cochlear Implant Questionnaire (NCIQ) [21] or the Patient Quality of Life Form and Index Relative Questionnaire Form (IRQF) [22] to measure HRQoL after cochlear implantation [18].

The aim of the present study was to prospectively assess the HRQoL in unilaterally or bilaterally hearing-impaired patients with or without a hearing aid following cochlear implantation. To assess the effect of a CI on functional outcomes and well-being, we used the NCIQ, Abbreviated Profile of Hearing Aid Benefit (APHAB), and Hearing Participation Scale (HPS). Patient data were examined prospectively using an exploratory, longitudinal study design. Questionnaires were completed before implantation and 3, 6, and 12 months after implantation.

## Materials and methods

### Ethical considerations

The ethics committee of the Medical Faculty at the University of Heidelberg granted permission to conduct the study (project no S-481/2009). The study was performed according to the Declaration of Helsinki on biomedical research involving human subjects. All patients were informed about the study aims and protocol, and participants were enrolled after giving informed written consent.

### Patient recruitment and patient data

All patients fitted with a cochlear implant at our department between 2011 and 2013 were considered for inclusion in the study. Implant systems were manufactured by MED-EL, Cochlear, and Advanced Bionics, and all implants were fitted by one of the authors (Ma. P.). Inclusion criteria were unilaterally or bilaterally deafness or severely hearing impairment and ≥ 18 years of age, and exclusion criteria were any medical condition requiring medication or surgical therapy and not giving consent to participate in the study. Surgical and clinico-pathological characteristics (age, gender, surgical report, implant systems, marital status, smoking habits, education, and professional activities) were recorded. Hearing performances were not included in our evaluation. All data were pseudonymized. For the investigations of the respective influencing factors on the questionnaire results, the patient cohort (*n* = 100) was divided into uni- (*n* = 57) and bilaterally (*n* = 43) deaf patients and into patients with (*n* = 54) or without hearing aids (*n* = 46).

### HRQoL assessment

Patients’ HRQoL was subjectively assessed using two established and validated questionnaires (NCIQ and APHAB) before implantation. In addition to the NCIQ and APHAB, the HPS and VAS questionnaires were used to assess QoL at 3, 6, and 12 months after implantation. The NCIQ is a standard questionnaire for assessing the QoL in patients fitted with CIs, and is reliable and sensitive to clinical changes [1, 8, 13, 21]. It comprises 60 questions divided into three general domains with respective subdomains: physical (basic sound perception, advanced sound perception, and speech production); psychological (self-esteem); and social (limited activity and social interaction [21]. Scores range from 0 (extremely poor) to 100 (excellent) [23]. The APHAB questionnaire is an abbreviated version of the Profile of Hearing Aid Benefit, which was originally designed to measure the benefit a patient gains from using a hearing aid. The APHAB comprises 24 questions, each with seven possible answers. Each question is answered twice - with and without a hearing aid. The questions are internally divided into four subscales: ease of communication (EC), background noise (BN), reverberation (RB), and aversiveness of noise (AV) [24]. A lower value indicates a better result [25]. The HPS is a shortened form of the Glasgow Health Status Inventory and includes 11 items covering three dimensions: self-esteem (questions 1–4 and 11), social handicap (questions 5–8), and hearing handicap (questions 9–11) [19]. Scores range from 0.00 to 1.00. A high score (maximum 55) indicates low impairment and a low score indicates high impairment or social handicap [26]. Satisfaction with the CI was assessed by the Visual Analogue Scale (VAS). Patients marked the subjective value applicable to them as a horizontal line on a scale from 0 (very unsatisfied) to 100% (very satisfied). The VAS has good validity [27], and is widely used in pain research and therapy.

### Statistical analysis

Statistical analysis was performed at the GESIS-Leibniz-Institute for the Social Sciences. Data were analyzed using the statistical software R (version 3.6.1). Principal component analysis (PCA) and exploratory factor analysis (EFA) were conducted using *psych*, *nFactor* and *FactoMineR* libraries. To determine the number of main components, graphical and non-graphical PCAs were used, including scree plots, eigenvalue analysis, parallel analysis, and optimal coordinates as suggested by Kaiser [28] and Cattell [29]. Both PCA and EFA are ways to simplify a particular set of data (in our case the genuine items that were measured for a particular index) by reducing the dimensionality of the initial data set. This is achieved by condensing the initial data into principal components that each contain variables of the data set that are highly correlated and therefore measure the same construct. These components then contain a linear combination of the related items/variables. The optimal number of components can be determined by the increment of the overall explained variation of the data by the newly formed orthogonal combination of the principal components [30]. We used this method to test whether our data fit the arrangement of subindices already established by the literature.

Sociodemographic characteristics were analyzed using generalized regression models. Metric variables are presented as means ± standard deviation, and categorical variables are presented as absolute numbers and percentages. Potential differences between groups (e.g., unilateral vs. bilateral hearing loss; hearing aid vs. no hearing aid) were examined using the Wilcoxon test for nonparametric data and Student’s test for parametric data. Differences in questionnaire scores between groups were determined using paired *t* test and one-way ANOVA. A *p* value less than 0.05 was considered statistically significant. Patient data were analyzed and compared. Each index and its subindices were graphically compared using R’s *ggplot2* package.

To determine the relatedness between the various questionnaire inventories, we performed the Pearson’s product–moment correlation test and the ${\chi }^{2}$ test for independence between each of the indices and time points.

## Results

### Patient cohort

A total of 100 deaf and severely hearing-impaired patients (54 females, 46 males) undergoing cochlear implantation were included in this prospective monocentric study. The average age at the time of implantation was 55.3 ± 16.9 years. Fifty-seven patients had unilateral deafness and 43 patients had bilateral deafness. Fifty-four patients used a hearing aid and 46 patients did not. The average duration of deafness was 213.2 ± 203.6 months (range 2–659). Eighty-five patients (85%) completed the 3-month follow-up, 65% completed the 6-month follow-up, and at 12 months, the dropout rate was approximately 41%, leading to 59% of remaining participants. Due to the small amount of initial data points, which is accompanied by a larger variance of estimates, an in-depth analysis of attritors was dismissed. However, Lugtig [31] argues that most reasons of attrition can be contributed to socio-demographic (e.g., age or gender), socio-psychological (e.g., a break in the habit of answering to the survey or absence of commitment), or (external) “shocks” such as health issues or moving. Hence, there is a possibility that aside of the negative impact of these dropouts on an estimator’s variance, it may further impact the (un-) biasedness of an estimator due to systematic reasons.

**Fig. 1:**
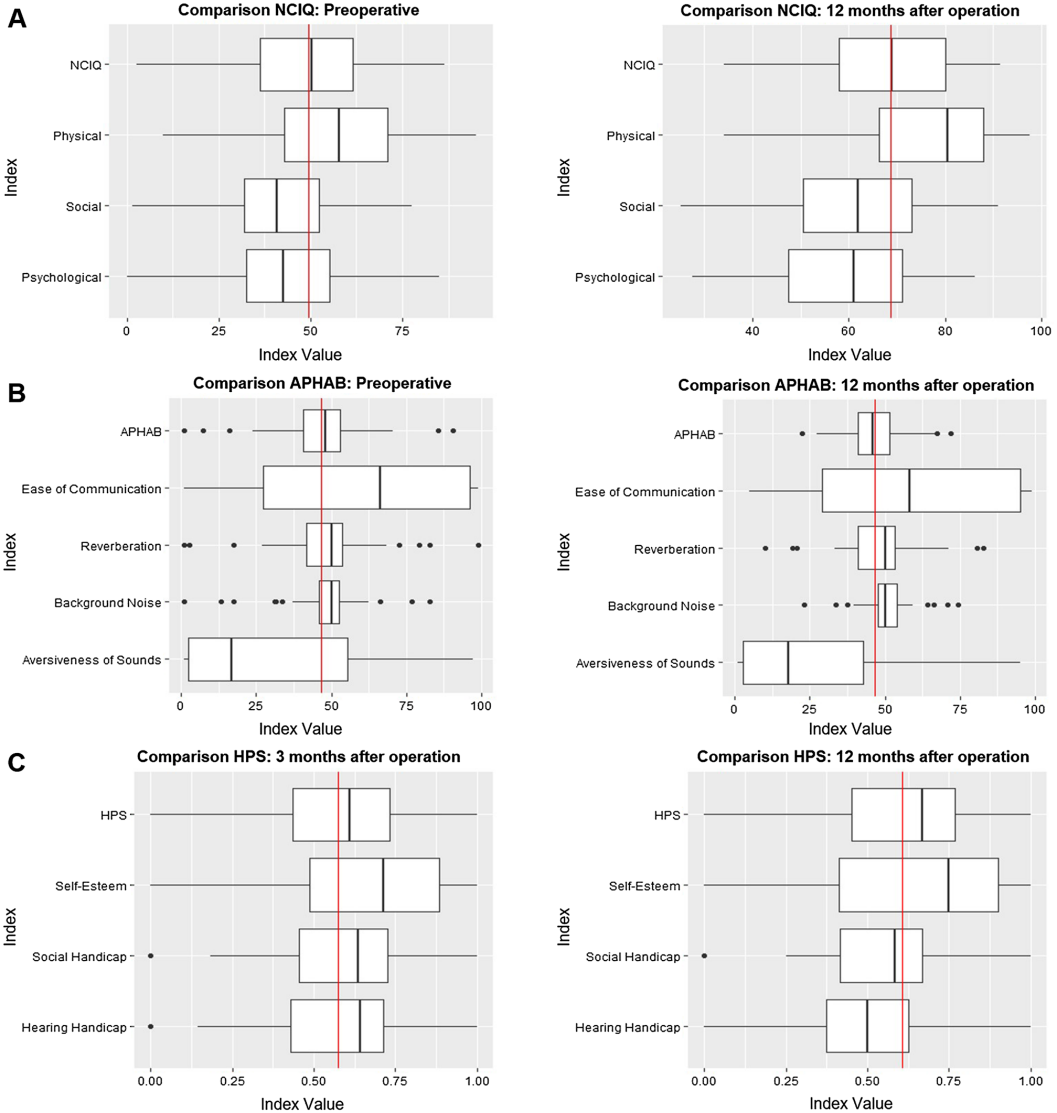
Box plot illustration of the used questionnaires. **a** Box plot illustration of the NCIQ total score and its three domains (physical, social, and psychological) at different time points (preoperative and 12 months after operation) of the total patient cohort (*n* = 100). The red line represents the overall mean NCIQ score. The bold line shows each distribution’s median, whereas the box represents the interquartile range. Dots resemble outliers. Mean NCIQ is displayed by the red line. **b** Box plot illustration of the APHAB total score and their four domains (EC, RV, BN, and AV) during the measurement times (preoperative and 12 months after operation) of the total patient cohort (*n* = 100). The red line represents the overall mean NCIQ score. The bold line shows each distribution’s median, whereas the box represents the interquartile range. Dots resemble outliers. Mean APHAB is displayed by the red line. **c** Box plot illustration of the HPS total score and their three domains (self-esteem, social, and hearing handicap) after implantation (3 months after operation and 12 months after operation) of the total patient cohort (*n* = 100). The red line represents the overall mean HPS score. The bold line shows each distribution’s median, whereas the box represents the interquartile range. Dots resemble outliers

Figure 1 and Table 1 illustrate the patient scores for the (sub)domains of each QoL instrument.

**Table 1:** Overview of the disease-specific questionnaire results of the total patient cohort (*n* = 100) over time

Disease-specific questionnaire	Before implantation	3 months after implantation	6 months after implantation	12 months after implantation	*p* value before implantation to 12 months after implantation	Absolute change after 12 months
Nijmegen cochlear implant questionnaire (NCIQ)						
Overall	49.35 ± 17.40	63.52 ± 14.76	67.03 ± 13.14	68.64 ± 13.75	**< 0.01**	+ 19.29
Physical	56.37 ± 20.48	70.55 ± 16.36	74.49 ± 13.53	76.21 ± 14.63	**< 0.01**	+ 19.84
Basic sound perception	48.11 ± 22.28	67.26 ± 19.34	72.88 ± 13.99	74.12 ± 15.64		+ 26.01
Advanced sound perception	70.33 ± 22.62	80.39 ± 17.95	81.89 ± 17.50	84.58 ± 16.95		+ 14.25
Speech production	50.68 ± 24.37	64.01 ± 19.75	68.69 ± 18.54	69.92 ± 19.07		+ 19.24
Psychological	43.82 ± 18.38	55.89 ± 17.56	59.45 ± 15.87	59.72 ± 15.71	**< 0.01**	+ 15.90
Self-esteem	43.82 ± 18.38	55.89 ± 17.56	59.45 ± 15.87	59.72 ± 15.71		+ 15.90
Social	41.84 ± 15.98	56.38 ± 15.47	59.50 ± 15.12	61.45 ± 15.78	**< 0.01**	+ 19.61
Activity limitations	42.05 ± 18.86	57.43 ± 17.77	60.16 ± 18.31	62.83 ± 18.67		+ 20.78
Social interactions	41.63 ± 15.38	55.32 ± 16.01	58.84 ± 13.81	60.08 ± 14.87		+ 18.45
Abbreviated profile of hearing aid benefit (APHAB)						
Overall	46.80% ± 13.03	46.16% ± 10.56	47.91% ± 11.63	46.57% ± 10.17	Without HA: 0.99 with HA: 0.24	− 0.23
Ease of communication (EC)	61.15% ± 33.94	57.91% ± 34.35	58.05% ± 34.42	60.26% ± 33.11	Without HA: 0.29 **with HA: 0.01**	− 0.89
Background noise (BN)	49.34% ± 11.62	50.41% ± 11.22	50.21% ± 10.69	50.66% ± 8.42	Without HA: 0.79 with HA: 0.56	+ 1.32
Reverberation (RV)	49.60% ± 15.16	49.29% ± 13.44	49.84% ± 12.01	48.83% ± 13.27	Without HA: 0.79 with HA: 0.72	− 0.77
Aversiveness of sounds (AV)	28.70% ± 28.99	26.84% ± 27.41	30.26% ± 28.08	26.46% ± 26.13	**Without HA: 0.02 **with HA: 0.56	− 2.24
Hearing participation scale (HPS)						
Overall	NA	0.58 ± 0.24	0.61 ± 0.24	0.61 ± 0.24	0.21*	+ 0.03
Self-esteem	NA	0.66 ± 0.26	0.66 ± 0.27	0.66 ± 0.27	0.75*	0
Social handicap	NA	0.57 ± 0.19	0.54 ± 0.18	0.54 ± 0.18	0.19*	− 0.03
Hearing handicap	NA	0.60 ± 0.21	0.49 ± 0.21	0.49 ± 0.21	0.004*	− 0.11
Visual Analogue Scale (VAS)						
Overall	NA	75.57 ± 20.36	77.26 ± 18.63	74.84 ± 21.66	0.64*	− 0.73

Values are mean and standard deviation. Significant changes are marked in bold. Unlike NCIQ and APHAB scores, the *p* values of HPS and VAS scores were calculated from the 3-month to 12-month follow-up and are indicated with an asterisk

*NA* not available, *HA* hearing aid

### VAS score

The postoperative VAS scores showing patient satisfaction were stable over time and did not differ between the follow-ups. VAS scores were not different between patients who used a hearing aid and those who did not. Bilaterally deaf patients had significantly higher VAS scores than unilaterally deaf patients over time (81.9 vs. 71.2 at 3, 6 and 12 months; *p* = 0.01), indicating higher levels of satisfaction. The degree of deafness (unilateral vs. bilateral) significantly influenced the VAS score at 3 months ($\widehat{\beta }=13.46$, *p* = 0.02), and age significantly influences the VAS score at 12 months ($\widehat{\beta }=0.48$, *p* = 0.04).

### Nijmegen Cochlear Implant Questionnaire (NCIQ)

As shown in Fig. 1a and Table 1, the overall NCIQ score significantly increased after cochlear implantation. The difference was highest in the basic sound perception subdomain and lowest in the advanced sound perception subdomain. Preoperative NCIQ scores were significantly different to the postoperative scores in the total cohort (*n* = 100). Uni- and bilaterally deaf patients only differed significantly preoperatively (54.3 vs. 42.8; *p* < 0.001) but not postoperatively. Wearing a hearing aid did not significantly affect the NCIQ score at any time point. When focusing on the three NCIQ (sub)domains, we could demonstrate that patients without a hearing aid had significantly higher scores in the physical domain before implantation (51.1 vs. 62.5; *p* < 0.01) and at 12 months follow-up (72.9 vs. 80.2; *p* = 0.05). Unilaterally deaf patients had significantly higher scores in the physical domain before implantation (63.9 vs. 46.4; *p* < 0.01) and 3 months after implantation (73.6 vs. 65.9; *p* = 0.03) than bilaterally deaf patients did. These findings suggest that unilaterally deaf patients who do not wear a hearing aid benefit less physically from the cochlear implantation than bilaterally deaf patients who wear a hearing aid do, because the scores for the physical domain remain mostly at baseline. Scores in the social and psychological domains were not significantly different between the groups (*p* > 0.05) at all points of measurement. Concerning the socio-demographic domain, whether hearing impairment was unilateral or bilateral only had a significant effect on the physical subscore before implantation ($\widehat{\beta }=-12.78$, *p* < 0.01). Professional activity affected the physical subscore 3 months after implantation ($\widehat{\beta }=-4.11$, *p* = 0.03). Scores in the social domain were significantly affected by graduation (preoperatively: $\widehat{\beta }=4.3$, *p* = 0.04), professional activity ($\widehat{\beta }=-3.63$, *p* = 0.05), and age ($\widehat{\beta }= -0.3$, *p* = 0.03) at 3 months after implantation. The duration of deafness significantly influenced the score in the psychological domain 6 months after implantation ($\widehat{\beta }=-0.02$, *p* = 0.04).

We used PCA and EFA to test the six subcategories of the three NCIQ domains as previously described [21], aiming to verify the established assumptions of the classification of subindices. The graphical and non-graphical approaches of PCA suggest a model composed of three components rather than six components. However, we performed an EFA for a six- and three-component model. We found that a three-component model only describes 39% of the index’s variance. Since the addition of three more components only explained a further 4% of the variance, the three-component model should be preferred. This makes PCA of utmost importance as ‘hypothesis generating’ tool creating a simple and clear principal component construct. The basic sound perception and speech production subcategories of the physical domain were loaded on factor 1, whereas the advanced sound perception subcategory of the physical domain fitted into factor 3. Factor 2 included scores from the social and psychological subdomains, which we collectively termed the socio-psychological domain.

### Abbreviated profile of hearing aid benefit (APHAB)

The APHAB score decreased over time, except for the BN subscale (Fig. 1b, Table 1). The preoperative and postoperative APHAB scores were not significantly different in patients who did not wear a hearing aid, whereas scores in patients who wore a hearing aid increased significantly after implantation (3 months: *p* = 0.056; 6 months: *p* < 0.01). Scores were significantly lower in unilaterally deaf patients than in bilaterally deaf patients at the 6-month follow-up (45.9 vs. 51.8%; *p* = 0.05), indicating that bilaterally deaf patients experience less postoperative benefit than unilaterally deaf patients do. Unilaterally deaf patients who did not wear a hearing aid had significantly lower scores in the EC subdomain before and after implantation (*p* < 0.01), indicating that a CI improved EC for those patients. Scores in the RV and BN subdomains were not significantly different between the groups at any time point (*p* > 0.05). Patients who did not wear a hearing aid had significantly higher scores in the AV subdomain before implantation (22.2 vs. 36.4%; *p* = 0.02), 3 months after implantation (20.6 vs. 35.0%; *p* = 0.02), and 12 months after implantation (19.4 vs. 34.1%; *p* = 0.03), indicating lower satisfaction for these patients. Bilaterally deaf patients had significantly lower scores in the AV subdomain at all time points (*p* < 0.01). The degree of impairment also affected scores in the EC subdomain at all time points in patients who did not wear a hearing aid (before: $\widehat{\beta }=23.49$, *p* < 0.01). Age influenced the APHAB score at 12 months after implantation in patients who wore a hearing aid ($\widehat{\beta }=0.49$, *p* = 0.05). Scores in the RV subdomain were influenced by marital status only in patients without a hearing aid before: ($\widehat{\beta }=5.79$, *p* = 0.04) and by graduation (before: $\widehat{\beta }=6.66$, *p* = 0.01) in patients with a hearing aid. Scores in the BN subdomain were influenced by gender, graduation, and professional activity before implantation in patients without a hearing aid, and by age and gender 3 months after implantation in patients with a hearing aid. Gender and degree of hearing impairment affected AV scores before and after implantation in patients without a hearing aid. In patients with a hearing aid, AV scores were affected by the degree of hearing impairment before and 3- and 6-month cochlear implantation. Gender affected AV scores at 12 months after implantation ($\widehat{\beta }=-19.68$, *p* = 0.01).

We verified the system described by Cox and Alexander [32] using PCA and EFA, which revealed a two-component model of APHAB, that significantly (*p* < 0.001) explained 59% of the total variance. Items 1–2, 4–7, 9–10, 12, 14–16, 18–19, 21, 23, and 24 were loaded on component 1, corresponding to EC, BN, and RV, while the rest of the items fitted into component 2 (similar to AV).

### Hearing participation scale (HPS)

HPS scores improved slightly over time and stabilized from 6 to 12 months after implantation (Fig. 1c, Table 1). The hearing handicap subscore significantly deteriorated from 3 to 12 months after implantation, indicating greater hearing impairment over time. Wearing a hearing aid or degree of hearing impairment did not affect the scores over time, and there were no differences in the three subscores between groups. Professional activity affected the HPS score at the 3-month follow-up ($\widehat{\beta }=-0.08$, *p* = 0.01).

PCA and EFA analyses revealed that the HPS score can be better explained by two components, which explain 45% of the total variance. The addition of a third component only explained a further 6% of the variance. Questions 1–4 and 8–11 were loaded to factor 1, which comprised questions on self-esteem and hearing handicap. These questions were recently summarized as an overall effect of the hearing handicap on QoL. Questions 5–7 were assigned to factor 2, largely in agreement with the pre-existing social handicap.

### Comparisons between the questionnaires

**Fig. 2:**
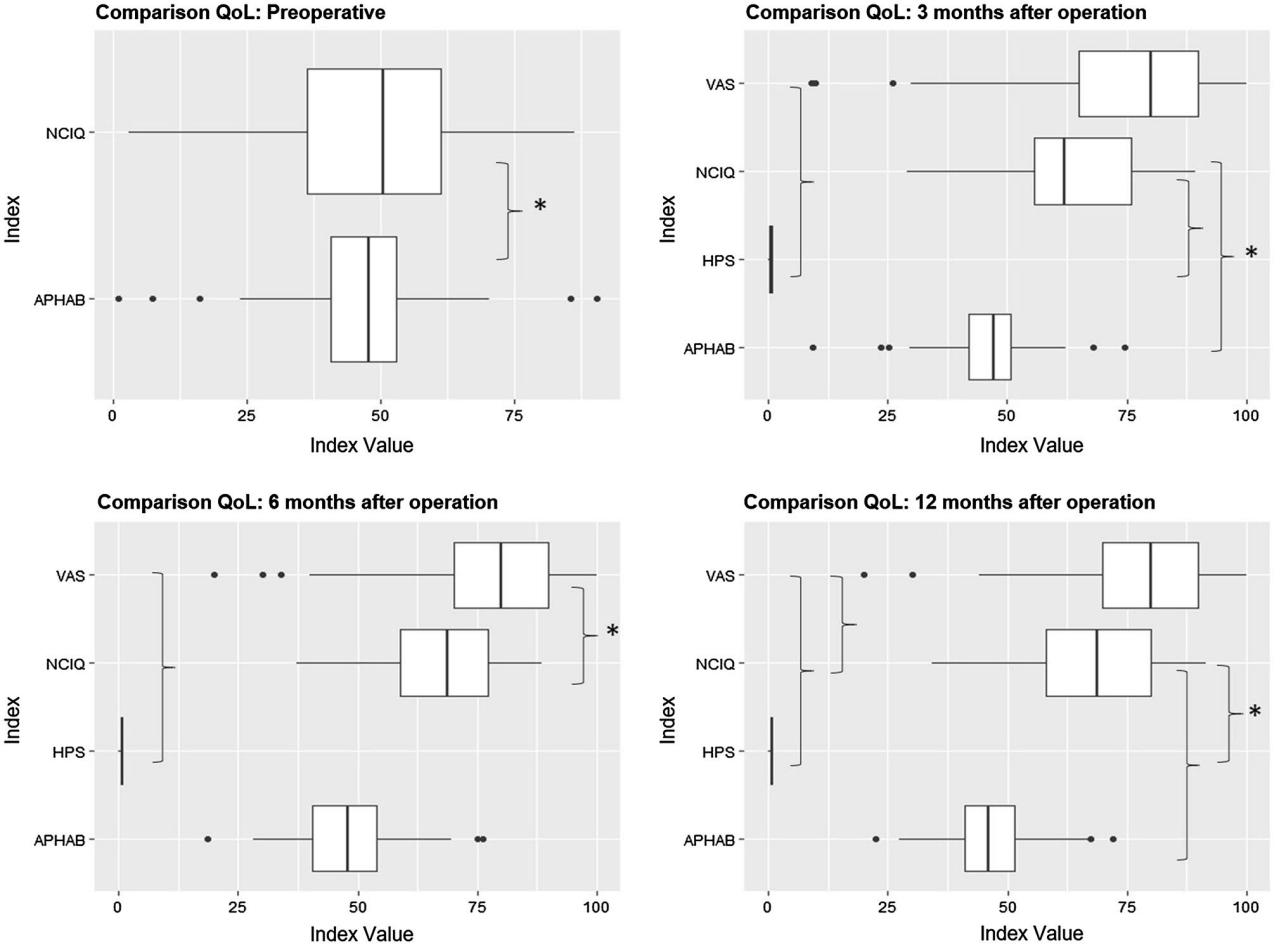
Box plot illustration of the correlation between the different, here selected questionnaires at different time points (preoperative, 3 months after operation, 6 months after operation, and 12 months after operation) of the total patient cohort (*n* = 100). Significant correlations are marked with brackets and an asterisk (*p < 0.05). A higher NCIQ score indicates better results, whereas a higher APHAB score indicates a worse result. High HPS scores indicate little impairment, whereas lower scores indicate greater impairment or social handicap

Pearson correlation coefficients showed a significant negative correlation between the APHAB and the NCIQ scores before implantation ($\widehat{\rho }= -0.22$, *p* = 0.03) and at 3 months ($\widehat{\rho }= -0.25$, *p* = 0.03) and 12 months ($\widehat{\rho }= -0.33$, *p* = 0.02) after implantation. The APHAB did not significantly correlate with the VAS and the HPS scores, but there was a significant correlation between the NCIQ and the HPS scores ($\widehat{\rho }= 0.29$, *p* < 0.01). NCIQ scores significantly correlated with VAS scores at the 6-month and 12-month follow-up ($\widehat{\rho }= 0.35$; $\widehat{\rho }= 0.31$, *p* < 0.01). There was also a significant correlation between HPS scores and VAS scores (Fig. 2).

## Discussion

Measuring the HRQoL is becoming more important in clinical and research settings [19]. To thoroughly assess QoL, both generic and disease-specific questionnaires should be used [12, 26, 33]. The use of generic instruments, such as the HUI3, is recommended [34] to provide the health-economic evaluations underpinning commissioning decisions in publicly funded healthcare systems [33]. As generic QoL measures capture a wide range of aspects in the health status of a person [35], they focus on conditions that may not be influenced by cochlear implantation and, therefore, they tend to be less sensitive [18] to evaluate subjective outcomes and QoL changes induced by cochlear implantation. Addressing this question was the main goal of our study, and that is why, we exclusively used disease-specific instruments. QoL measurements in patients fitted with CIs can be compared systematically between patient groups. In this study, we compared unilaterally and bilaterally hearing-impaired patients and patients with and without a hearing aid using the NCIQ, APHAB, and HPS questionnaires to assess the patients’ perception of their QoL before and after cochlear implantation.

As described in diverse studies, the NCIQ, developed by Hinderink et al. to encompass not only hearing and speech production but also the psychological and social domains [21], measured important improvements in the NCIQ total score as well as in all six domains between the CI-pre and CI-post assessments [1, 8, 9, 13, 21, 36]. In line with the previous studies, we observed the largest improvement in the basic sound perception subdomain of the physical domain [13, 21] followed by the activity limitations subdomain of the social domain. This is understandable, because progressive hearing loss significantly affects a person’s social life. For example, in social situations where lots of people are speaking, a person with hearing impairment will find it challenging to follow simple conversations [37], creating a feeling of profound social isolation and reduced QoL [38, 39]. In agreement with the findings of Hinderink et al. [21], we observed that a CI not only improves hearing and speech production, but also improves self-esteem, daily activities, and social functioning. Contrary to the results of Hinderink et al. [21] and Krabbe et al. [13], we observed the lowest improvement in the advanced sound perception subdomain (physical subdomain). This may be because a single CI can improve speech recognition and fulfill a person’s basic auditory needs, but bilaterally deaf people still find it difficult to listen in environments with high background noise and multiple speakers. The inability to perceive the directionality of sound makes this even more difficult [40]. However, the NCIQ subscores revealed physical benefits in bilaterally deaf patients who wear a hearing aid after unilateral cochlear implantation, emphasizing that a CI may improve QoL [37].

Based on the recommendation of Kitterick et al. [33] for assessing hearing-related quality of life, we also used the APHAB to quantify hearing difficulties in everyday listening situations [32]. In accordance with the findings of Zwartenkot et al. [25], we found that APHAB scores improved over time, except for BN, indicating a positive effect of cochlear implantation on HRQoL. As described by Zwartenkot et al. [25], the deterioration of BN could be due to progressive hearing loss (similar to slowly progressing presbyacusis). We showed that a unilateral CI significantly improved EC for unilaterally deaf patients who do not wear a hearing aid. However, we did not see any differences in the ability to hear in reverberate situations or in situations with background noise between the groups. At the moment, we cannot explain this finding. Bilaterally deaf patients who wore a hearing aid had significantly more aversiveness to sounds over time, which indicates negative reactions to environmental sounds [25]. Consequently, having cochlear implants in both ears may help people to hear better in noisy places and find where sounds came from [37]. Studies have shown that QoL improves more after bilateral cochlear implantation [1] than after unilateral cochlear implantation, and that EC, BN, and RV are improved [41, 42]. This explains why patients fitted with unilateral CIs generally express a desire for bilateral implants [37]. However, the VAS scores reported here show that bilaterally deaf patients were significantly more satisfied with their CIs than unilaterally deaf patients were. Generally, there is an ongoing global discussion on whether or not bilateral cochlear implantation should be standard care for bilateral deafness. In this context, the study group of Ramakers et al. reported on the positive effect of bilateral cochlear implantation on preoperative tinnitus complaints, which should be taken into account when counseling a patient [43]. Taken together, these findings show that cochlear implantation is a successful treatment for improving QoL in deaf patients, and that both the NCIQ and the APHAB seems to be good hearing instruments in adults with a unilaterally and bilaterally severe to profound sensorineural hearing loss. Hearing impairment can also be evaluated using the HPS questionnaire. In agreement with the findings of Hogan and Hawthorne [10, 44], the total HPS score improved over time and stabilized between 6 and 12 months after implantation. However, contrary to their findings, we could not confirm an improvement in the subscores. Instead, we observed that the hearing handicap subscore significantly deteriorated between 3 and 12 months after implantation, indicating greater hearing impairment over time. This could be a natural consequence of aging.

The heterogeneity of our results may be explained by the different questionnaires used, each of which uses different factors to assess QoL. Standardized CI-specific HRQoL instruments would help here. In the present study, we have shown that the NCIQ is a good first-line questionnaire for assessing QoL in deaf patients after cochlear implantation. NCIQ scores correlated well with those of other QoL instruments, and the NCIQ has a plausible three-domain factor classification.

With the aim to either verify the established (sub)domain classification of the questionnaires or suggest possible changes that may further help to reduce the high-dimensionality of the questionnaires into few (mostly) uncorrelated components, we used PCA and EFA. These proposed novel assignments of items to particular components. The NCIQ score can be explained by three components based on the original division by Hinderink et al. [21] but with different assignments. These three components are physical, physical advanced, and socio-psychological. For the APHAB developed by Cox and Alexander [32], the PCA revealed a two-factor solution, with EC, RV, and BN comprising factor 1 and AV comprising factor 2. The original three HPS components described by Hawthorne and Hogan [26] can be better explained by two components, which we call overall effect of the hearing handicap on QoL and social handicap.

We observed only a weak socio-demographic influence on the questionnaire scores. Similar to the findings of Bess et al. [45], the degree of hearing loss influenced the level of physical disability measured by the NCIQ. We also showed that the degree of hearing impairment affected the EC and AV subdomains of the APHAB as well as the VAS scores. We also saw no clear effect of age on QoL scores, so included patients from 18.7 to 87.4 years of age rather than focusing on elderly patients like in the studies of Dalton et al. [46] and Nordvik et al. [23]. Our finding that age at implantation is not directly linked to HRQoL is supported by the other studies [8, 47, 48].

There are some limitations to the present study. Assessing QoL is limited by the survey designs, which do not consider cognitive or communicative abilities and whether the patients can assess their own functional limitations. A further criticism is that patient satisfaction is extremely subjective [49], and results can be biased by context effects, such as the current mood. Our attrition rate of 41% after 12 months introduces the possibility of selection bias [19], which may have affected the results. The impact is more obvious for the estimators’ variances, since these will be increased due to the reduction in sample sizes. The impact may, therefore, produce false negative results, especially when regarding the significance of characteristics influencing an index’s outcome. However, due to the magnitude of significance in our findings, it may be assumed that this particular effect has not occurred. It is, therefore, more likely that we neglected influences that would have been significant, given a larger sample size. To mitigate such problems, we opted for a variance estimation based on Monte Carlo simulations for our comparisons. Nevertheless, since the Monte Carlo variance is also an estimate and cannot reduce the impact of a small sample size in its entirety, the generalizability of these results is limited. Another important limitation, however, is that the attrition may have been systematic and may be the result of socio-demographic or socio-psychological characteristic as well as extrinsic shocks. This may then in turn also have influenced the biasedness of our estimates. Given the limited data points available, we can only assume that our findings would hold true when reproduced with a larger sample size.

Furthermore, the prospective study design may have introduced recalibration, reprioritization, and reconceptualization response shifts in the post-test phase [50]. However, response shifts are probably minimal in CI patients, because they re-experience deafness whenever they remove their speech processor.

Our study also has some strengths. Most previous studies have assessed QoL retrospectively, after cochlear implantation and with low patients’ numbers and measurement time points [8, 9, 13, 21]. In contrast, our study has a prospective study design, uses three disease-specific questionnaires, and measures QoL before implantation and at three time points after implantation. Further research is needed to explore long-term changes in QoL after cochlear implantation.

## Conclusions

Assessing HRQoL after cochlear implantation using standardized and validated disease-specific questionnaires is an important part of audiological diagnostics. Results from standardized QoL questionnaires can be compared between different studies to monitor the effects of technical improvements to cochlear implantation. We suggest that the NCIQ be used as a first-line questionnaire to assess psychological, social, and physical domains as these results correlated significantly with those of different questionnaires.

## Data Availability

The datasets generated during and/or analyzed during the current study are available from the corresponding author on reasonable request.
